# Isolation of *Malassezia* spp. in HIV-positive patients with and without seborrheic dermatitis^☆^^☆☆^

**DOI:** 10.1016/j.abd.2019.09.012

**Published:** 2019-09-30

**Authors:** Gabriela Moreno-Coutiño, Carlos D. Sánchez-Cárdenas, Yesenia Bello-Hernández, Ramón Fernández-Martínez, Sara Arroyo-Escalante, Roberto Arenas

**Affiliations:** a Mycology Department, Dr. Manuel Gea González General Hospital, Delegación Tlalpan, Mexico City, Mexico; b Medical Student, Dr. Manuel Gea González General Hospital, Delegación Tlalpan, Mexico City, Mexico; c Research Department, Dr. Manuel Gea González General Hospital, Delegación Tlalpan, Mexico City, Mexico; d Head of the Mycology Department, Dr. Manuel Gea González General Hospital, Delegación Tlalpan, Mexico City, Mexico

**Keywords:** AIDS-related opportunistic infections, Dermatitis, seborrheic, *Malassezia*, Yeasts

## Abstract

**Background:**

*Malassezia*, a skin saprophyte, is frequently isolated from patients with seborrheic dermatitis, which is one of the most common dermatoses in HIV-infected patients. Its role in pathophysiology has not been defined.

**Objective:**

To determine whether patients living with HIV and seborrheic dermatitis have more *Malassezia* than those without seborrheic dermatitis.

**Method:**

This is an descriptive, observational, prospective cross-sectional study to which all adult patients living with HIV that attend the infectious disease outpatient clinic at the Dr. Manuel Gea González General Hospital were invited. Patients presenting with scale and erythema were included in Group 1, while patients without erythema were included in Group 2. Samples were taken from all patients for smear and culture.

**Results:**

Thirty patients were included in each group. All patients with seborrheic dermatitis had a positive smear, with varying amounts of yeasts. In the control group, 36.7% of patients had a negative smear. The results are statistically significant, as well as the number of colonies in the cultures.

Study limitations The study used a small sample size and the subspecies were not identified.

**Conclusions:**

Patients with clinical manifestations of seborrheic dermatitis have larger amounts of *Malassezia*. Further studies need to be performed to analyze if the greater amount is related to imbalances in the microbiota of the skin.

## Introduction

*Malassezia* spp. yeasts are saprophytes, part of the skin's normal flora, which can also be identified in some pathologic conditions that vary in severity from superficial mycosis to fungemia.

*Malassezia* spp. have been identified as the causative agents of pityriasis versicolor and some cases of folliculitis. However, their role in seborrheic dermatitis (SD) remains controversial and, although many data support the inflammatory origin of the dermatosis, its relation with *Malassezia* spp. remains both undeniable and unexplained.^1^

SD is an inflammatory dermatosis that affects mainly the scalp, and less frequently the face and trunk. Scales, erythema, and itching are the most common findings. It affects around 3% of the world population, predominates in males, and is much more frequent in association with Parkinson's disease and HIV infection, with an incidence of up to 80%. As it is particularly frequent in patients living with HIV, SD is sometimes considered a marker of the infection and can aid in early diagnosis.^2^

The physiopathology of SD has not been completely elucidated. However, several factors have been considered as possible triggers for the disease, such as androgen-dependent sebaceous glands, male sex, seasonal factors, sleep deprivation, infection, and stress. SD activity is characterized by a cyclic behavior of remissions and exacerbations.^3^

The diagnosis is supported by clinical manifestations, and no biopsies or additional studies are necessary. Cultures are not routinely done, since *Malassezia* spp. does not grow easily in conventional media. However, modified Dixon agar, a lipid-enriched culture, allows the isolation of the yeast with a success rate of 90%.^4^

Treatment options are broad and include keratolytics, steroids, and antifungals, all showing acceptable response. However, based on the relapsing nature of the disease, the authors do not recommend using steroids as the first line medication. However, ketoconazole has the advantage of being an antifungal that reduces the amount of *Malassezia* on the skin, but, more importantly, it has an anti-inflammatory effect that is very effective in SD control.^5^

The aim of this study was to quantify *Malassezia* yeasts (colony forming units, CFUs) in a population living with HIV, with and without clinical manifestations of seborrheic dermatitis. It is believed that these results will contribute to the body of knowledge on the role that *Malassezia* spp. plays in SD.

## Methods

This was an observational, descriptive, prospective, cross-sectional study. All adult patients living with HIV who attended the infectious disease outpatient clinic at the Dr. Manuel Gea González General Hospital were invited to participate. Those that accepted all signed an informed consent previously approved by the ethics and investigation committee of the hospital. Data such as age and gender were recorded, and patients were physically examined for clinical signs of SD on the face. Patients who presented with scaling and erythema were included in Group 1 and patients without those symptoms were included in Group 2. Patients who had received topical or systemic antifungals over the past six months were excluded.

The authors applied a drop of saline solution on a No. 20 scalpel blade and carefully scraped the glabellar area, in an atraumatic manner, to obtain a sample of epidermal cells and microbiota. The material was divided into two portions, half for methylene blue staining and half for a culture in m-Dixon agar medium incubated at 37 °C. The cultures were checked every 72 h for fungal growth during two weeks. After that, a direct exam with lactophenol cotton blue was performed in order to count the number of colonies.

For the smear staining, the sample was oven dried and 96% ethyl alcohol and direct heat were used for fixation. Finally, methylene blue was applied for 1 min and then washed. The authors used a 100× light microscope with oil immersion to observe the yeasts at 40× magnification. The results were scored as follows: 1–5 yeasts per field = +; 6–10 = ++; >10 = +++.

Fisher's exact test and SPSS v. 23.2 were used for the statistical analysis.

## Results

Sixty patients were included in this study, 30 in each group. Males predominated in both groups (90%), with an age range of 18–79 years, and an average of 35.4 years (±12.4 SD). In the first group, there were 28 men (93.4%) and two women (6.6%), with an average age of 32.7 years within a range of 18–79 years. In the second group, there were 26 men (86.7%) and four women (13.3%), with an average age of 38.2 years within a range of 23–75 years (Table 1). In the SD group, 80% of the patients had not started the antiretroviral therapy, whereas the majority of the non-affected group (73%) was on the medication. Most patients with SD had a CD4 cell count between 200 and 499 compared to 60% of the group without SD, where the CD4 cell count was higher than 500 (Table 2).

**Table 1:** Demographic data of recruited patients.

Group	Gender		Age range	
	Male *n* (%)	Female *n* (%)	Range	Average
HIV+ patients with SD (*n* = 30)	28/93 (4%)	2/6 (6%)	18–79	32.7
HIV+ patients without SD (*n* = 30)	26/86 (7%)	4/13 (3%)	23–75	38.2

SD, seborrheic dermatitis.

**Table 2:** Comparison of HIV treatment and CD4 cell counts in HIV+ patients with SD and HIV+ patients without SD.

	HIV patients with SD, *n* (%)	HIV patients without SD, *n* (%)
*ARV treatment*		
With ARV	6 (20%)	22 (73%)
Without ARV	24 (80%)	8 (27%)

*CD4 cell count (cells/μL)*		
>500 CD4	6 (20%)	18 (60%)
200–499 CD4	15 (50%)	8 (27%)
<200 CD4	9 (30%)	4 (13%)

*Middle range in CD4 count cells*	423.6	538.0

SD, seborrheic dermatitis; ARV, antiretroviral; CD4, cluster of differentiation 4.

In this study, all patients with SD (Group 1) had positive smear results for *Malassezia* spp. with the following frequencies: nine tests (30%) with low yeast concentration (+), 14 (46.7%) with moderate yeast concentration (++), and seven (23.3%) with abundant yeast concentration (+++). The group of patients without SD (Group 2) had positive smear results for *Malassezia* spp. with the following frequencies: ten tests (33.3%) with low yeast concentration (+), seven (23.3%) with moderate yeast concentration (++), and two (6.7%) with abundant yeast concentration (+++). Eleven patients (36.7%) had negative smear results for *Malassezia* spp. (Table 3).

**Table 3:** Comparison of PAS-positive smear for *Malassezia* spp. between HIV+ patients with and without SD.

	PAS-positive smear for *Malassezia* spp.	
	HIV patients with SD, *n* (%)	HIV patients without SD, *n* (%)
PAS+	9 (30%)	10 (33.3%)
PAS++	14 (46.7%)	7 (23.3%)
PAS+++	7 (23.3%)	2 (6.7%)
PAS−	0 (0%)	11 (36.7%)

SD, seborrheic dermatitis.

A significant statistical difference (*p* = 0.001) was found in the number of yeasts identified in stained smears from both groups, with predominance in patients with SD.

Regarding *Malassezia* spp. growth in the group of HIV patients with SD, 23 (76.7%) cultures were positive and seven (23.3%) were negative, with a yeast load range of 10–70 CFUs. In the group of patients without SD, 15 (50%) cultures were positive and 15 (50%) were negative, with a yeast load range of 2–35 CFUs.

The authors classified the cultures into four categories according to CFU: negative cultures with zero CFUs, cultures with 1–25 CFUs, cultures with 26–50 CFUs, and cultures with 51–75 CFUs. The frequency order for patients with SD was: seven cultures (23%) with zero CFUs, 11 (37%) with 1–25 CFUs, nine (30%) with 26–50 CFUs, and three (10%) with 51–75 CFUs. The frequency order for HIV+ patients without SD was 15 cultures (50%) with zero CFUs, 13 (43%) with 1–25 CFUs, two (7%) with 26–50 CFUs, and no cultures (0%) with 51–75 CFUs (Table 4).

**Table 4:** Comparison of CFU growth of *Malassezia* spp. between HIV+ patients with and without SD.

	CFU growth of *Malassezia* spp.	
	HIV patients with SD, *n* (%)	HIV patients without SD, *n* (%)
0 CFUs	7 (23%)	15 (50%)
1–25 CFUs	11 (37%)	13 (43%)
26–50 CFUs	9 (30%)	2 (7%)
51–75 CFUs	3 (10%)	0 (0%)

SD, seborrheic dermatitis; CFU, colony forming unit.

A significant statistical difference (*p* < 0.001) was found in both groups, with a predominance in patients with SD.

The average *Malassezia* spp. count was determined in the two groups: *n* = 37.67 in HIV+ patients with SD and *n* = 23.33 in patients without SD. After performing the Mann–Whitney *U*-test, the results showed a significant difference (*p* = 0.012) between both groups, showing that HIV+ patients with SD have an increased *Malassezia* spp. growth rate in comparison with HIV+ patients without SD.

## Discussion

As a disease that mainly affects visible areas such as the face and scalp, besides the discomfort caused by itching, the social impact of seborrheic dermatitis is relevant and can play an important role in quality of life. It is also important to remember that SD, considered an early sign of cellular immunosuppression, can be a disease marker in the HIV-infected population.^6^

A study that analyzed CFU data in patients living with HIV with and without SD compared to HIV-negative controls found no difference in the number of yeasts between the groups. The authors concluded that, apparently, individual immune response is what determines the clinical manifestations.^6^, ^7^, ^8^, ^9^

In comparison, the present results showed that *Malassezia* spp. can be isolated both from skin with and without seborrheic dermatitis in patients living with HIV, the only difference being the yeast load.

These results are similar to a report that compared HIV-positive and HIV-negative patients without skin lesions, which found a 94% growth rate of *Malassezia*. The difference between the groups was also the greater CFU count in HIV-positive patients.^7^

Although some authors still consider SD as an infectious disease, its inflammatory origin becomes more evident every day.^10^

However, the results from this study and from some other studies somehow strongly relate SD to the presence of *Malassezia* spp. Probably, the greater yeast load is secondary to the increase in sebum production. Thus, an active multiplication of yeasts is easily observed. As these fungi hydrolyze human sebum, the metabolites cause keratinocytes to liberate pro-inflammatory cytokines, completing the inflammation cycle.^11^

It is also possible that the overpopulation of *Malassezia* may harm the skin's microbiota balance, which could explain the importance of cellular immunity in SD, being more frequent in immunocompromised patients.

## Conclusion

Although the role of *Malassezia* is still not fully understood, apparently, the quantity of these yeasts is relevant for the development of symptomatic seborrheic dermatitis. Therefore, the key elements for further investigation should include lipid quantity and quality in affected and non-affected patients. Also, a further metagenomic analysis of the skin microbiota in health and disease (SD) for the comparison of the species (bacterial and fungal) present in such cases should be useful to further comprehend SD and its pathogenesis.^12^

## Key messages: what's new?

*Malassezia spp*. can be equally isolated from patients living with HIV/AIDS either with or without clinical manifestations of SD.

There is a statistical difference in the amount of yeasts in patients with and without seborrheic dermatitis.

The increased sebum production these patients present may predispose to an overload of *Malassezia* that subsequently alters the skin microbiota balance.

## Funding

None declared.

## Author's contribution

Gabriela Moreno-Coutiño: Approval of the final version of the manuscript; conception and planning of the study; elaboration and writing of the manuscript; intellectual participation in the propaedeutic and/or therapeutic approach of the studied cases.

Carlos D. Sánchez-Cárdenas: Statistical analysis.

Yesenia Bello-Hernández: Obtaining, analyzing and interpreting the data.

Ramón Fernández-Martínez: Statistical analysis.

Sara Arroyo-Escalante: Conception and planning of the study.

Roberto Arenas: Approval of the final version of the manuscript.

## Conflicts of interest

The authors declare no conflicts of interest.
